# Realizing Universal Health Coverage in East Africa: the relevance of human rights

**DOI:** 10.1186/s12914-017-0128-0

**Published:** 2017-08-03

**Authors:** Alicia Ely Yamin, Allan Maleche

**Affiliations:** 1 Law and Global Health, Harvard TH Chan School of Public Health, Boston, USA; 2 UN Secretary General’s Independent Accountability Panel for the Global Strategy (EWEC), New York, USA; 3 Georgetown University Law Center, 600 New Jersey Ave. NW, Washington, DC 20001 USA; 4 Kenya Legal & Ethical Issues Network (KELIN), Geneva, Switzerland

**Keywords:** Universal Health Coverage, East Africa, Human rights, Health systems, Fair financing, Priority-setting, Accountability, Participation

## Abstract

Applying a robust human rights framework would change thinking and decision-making in efforts to achieve Universal Health Coverage (UHC), and advance efforts to promote women’s, children’s, and adolescents’ health in East Africa, which is a priority under the Sustainable Development Agenda. Nevertheless, there is a gap between global rhetoric of human rights and ongoing health reform efforts. This debate article seeks to fill part of that gap by setting out principles of human rights-based approaches (HRBAs), and then applying those principles to questions that countries undertaking efforts toward UHC and promoting women’s, children’s and adolescents’ health, will need to face, focusing in particular on ensuring enabling legal and policy frameworks, establishing fair financing; priority-setting processes, and meaningful oversight and accountability mechanisms. In a region where democratic institutions are notoriously weak, we argue that the explicit application of a meaningful human rights framework could enhance equity, participation and accountability, and in turn the democratic legitimacy of health reform initiatives being undertaken in the region.

## Background

Lagging efforts to improve women’s, children’s and adolescents’ health have been a particular focus of development efforts in East Africa.^1^ The Sustainable Development Goals (SDGs) contain several health-related targets, of which promoting women’s and children’s health as well as Universal Health Care (UHC) attainment are critical aims [1]. The inclusion of UHC within the SDGs is a remarkable shift from previous health promotion efforts and has gained global support including from the World Health Organization (WHO) [2, 3]. The SDG targets are further elaborated upon in the 2015 UN Secretary General’s Global Strategy on Reproductive, Maternal, Newborn, Children’s and Adolescents’ Health (“Global Strategy”), which provides a roadmap to advancing the health of women, children and adolescents. Together, the SDGs and the Global Strategy will inevitably deeply influence financing, policy-making, and programming for efforts to achieve UHC, and promote the health of women, children and adolescents in East Africa. The Global Strategy makes explicit mention of human rights and there has been substantial rhetoric from global leaders [4, 5] about the need to use human rights-based approaches, including recognition of the right to health, to achieve the Global Strategy as well as the target related to UHC. Yet, there continues to be a gap between this rhetoric and what occurs on the ground. This article will focus on a few issues that countries will necessarily face in the context of advancing the Global Strategy and achieving UHC, and in signaling how applying human rights principles has the potential to enhance equity, accountability and participation.

The right to health and human rights principles are defined in international human rights law and are also enshrined in treaties that the countries of the East African Community (EAC)^2^ have ratified, and Kenya has also embedded in its 2010 constitution [6–11]. The right to health “requires that health-care goods, services and facilities be available in adequate numbers; financially and geographically accessible, as well as accessible on the basis of non-discrimination; acceptable, that is, respectful of the culture of individuals, minorities, peoples and communities and sensitive to gender and life-cycle requirements; and of good quality” [7]. UHC is a crucial aspect of realizing the right to health, but “not all paths to universal health coverage are consistent with human rights requirements” [5, 7, 10, 12].

The importance of human rights is increasingly cited in relation to health in the development agenda [7]. The UN Secretary General’s Global Strategy on Reproductive, Maternal, Newborn, Children’s and Adolescents’ Health (Global Strategy), a strategy to realizing the right to health for women, children and adolescents, is explicitly rooted in a human rights framework [13]. The 2016 report of the UN Secretary General’s Independent Accountability Panel (IAP) on the Global Strategy, created to ensure accountable implementation of the Global Strategy and on which one of these authors sits (self-identifying reference), even sets out an accountability framework based on human rights law [14].^3^ Yet, there remains a disconnect between this global rhetoric and the national realities of health reform initiatives in the region.

**Fig. 1:**
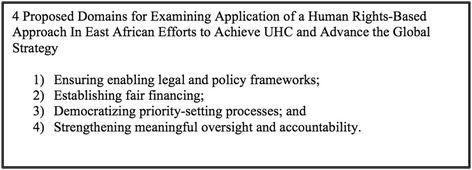
Four proposed domains

A human rights-based approach (HRBA) goes beyond the right to health and utilizes “human rights standards contained in, and principles derived from, the Universal Declaration of Human Rights and other international human rights instruments” to guide analysis and policy, legislation, programming, and evaluation and monitoring [8, 9, 15–18]. “Among [the] human rights principles are: universality and inalienability; indivisibility; interdependence and inter-relatedness; non-discrimination and equality; participation and inclusion; accountability and the rule of law” [8, 9, 15, 16]. A human rights-based approach also “contributes to the development of the capacities of ‘duty-bearers’ to meet their obligations and/or of ‘rights-holders’ to claim their rights” [8, 9, 15–18]. As Sanghera et al. explain in respect of the new Global Strategy: “A human rights based approach is based on accountability and on empowering women, children, and adolescents to claim their rights and participate in decision making, and it covers the interrelated determinants of health and wellbeing. [It] promotes holistic responses, rather than fragmented strategies, and requires attention to the health needs of marginalised and vulnerable populations [13].” In this debate article, we set out how meaningfully applying these principles of human rights would change thinking and decision-making in efforts to achieve UHC and other targets related to the Global Strategy in East Africa in four specific domains: ensuring enabling legal and policy frameworks; establishing fair financing; democratizing priority-setting processes; and strengthening meaningful oversight and accountability [19, 20]. These four domains are derived from international human rights law relating specifically to the right to health [8–11], principles of international human rights law more broadly [8, 9, 15, 16], interpretative guidance on aspects of the right to health [7, 21], and inter-governmental guidance on applying a human rights-based approach to policy, legislation and programming [16]. In a region where democratic institutions are notoriously weak, we argue that the application of a robustly understood human rights based approach (HRBA) could enhance equity, transparency and accountability, and in turn the legitimacy of health reform initiatives being undertaken in the region (Fig. 1).

## Context

In addition to having fragile democratic institutions, the East African region has some of the worst health statistics in the world, as well as some of the highest rates of income poverty [22, 23]. For example, WHO estimates maternal mortality ratios (MMRs), which reflect the functioning of health systems as well as the status of women in society, at 710 per 100,000 live births for Burundi, Kenya at 510, Rwanda at 290, Uganda at 343 and Tanzania at 398, compared to the global average of 221 in 2015 [24]. The interventions that might address these elevated MMRs, including skilled birth attendance (SBA), remain low in across the region, and as low as 43% in Kenya [25]. Furthermore, not only do other basic indicators of health system functioning such as infant mortality rates also remain high, and rates of non-communicable diseases are exponentially growing in the region while health systems struggle to address basic health conditions [26, 27]. Underpinning these dismal statistics and contributing to unmet population needs is the lack of a skilled, trained health care workforce that enjoys decent work standards and basic labor protections. The region experiences the most severe needs-based shortage of health care workers in the world, with only 1.9 total health care workers for every 1000 population [28]. WHO has forecasted this shortage to worsen between 2013 and 2030 [13].

As elsewhere, health systems in East Africa reflect historical and economic patterns of privilege, as well as social values [29]. Formal health systems in the region were established as part of colonial governance, with an enormous role of parochial institutions delivering health care as charity [30, 31]. These institutions still provide over 50% of care in the region [32]. Beginning in the 1980s and 1990’s, HIV/AIDS took an enormous toll on the region [33]. Vertical programs such as the United States President’s Emergency Plan for AIDS Relief (PEPFAR) or the Global Fund to fight AIDS, Tuberculosis and Malaria, as well as additional vertical programs targeted at other Millennium Development Goal (MDG) indicators, such as skilled birth attendance, did little to improve overall systems’ functioning, given that background conditions, including infrastructure and referral networks, were deeply unequal and fragile [34]. Along with narrow, targeted programs, the MDGs encouraged a technocratic, “apolitical” view of development, focused on efficiency of narrow outcome measures [35, 36]. Moreover, by 2010, the UN acknowledged that there was a lack of accountability in many countries’ efforts to meet MDGs 4 and 5, relating to maternal and child health, respectively, and the UN Secretary General launched the first Global Strategy on Women’s and Children’s Health, which was then expanded and made more robust in 2015 with the second Global Strategy on Women’s, Children’s and Adolescents’ Health to accompany the SDGs [37–39].

East Africa is rightly a focus for development partners concerned that weak health and demographic policies will hinder possibilities for economic growth. Health is also a priority area for regional cooperation among the five member states of the EAC. However, we argue here that technocratic approaches alone are unlikely to shift the underlying political determinants of health that hamper both the region’s development, and the possibility for the health system to perform the functions it should in the construction of inclusive citizenship [40]. The rest of this article sets out four ways in which applying a robust human rights framework would change thinking and decision-making about health reform on the path to UHC, and in turn in advancing women’s, children’s and adolescent’s health.

## Enabling legal and policy frameworks

Health systems are inexorably governed by legal and policy frameworks, despite too little attention being paid to these in conventional “systems thinking” [41, 42]. Indeed, legal frameworks function as social—and political—determinants of health more broadly by establishing the parameters of people’s entitlements, the responsibilities of different levels of government and the regulation of private actors, among other things. Laws structure the institutions that provide goods and services. Laws and policies also establish social norms that invariably affect health (e.g., criminalization of sodomy or IV drug use), which reflect narratives of equal concern and respect, or do not [20].

The new Global Strategy explicitly calls for multi-sectoral approaches in planning [43]. In that context, applying an HRBA first makes the legitimacy of the normative framework for health directly relevant to health reforms. An HRBA requires situational analyses with respect not just to epidemiological or demographic conditions, but also laws, when health sector reforms are undertaken or health policies/directives are adopted. Second, HRBAs provide criteria, including fundamental commitments to dignity (e.g., bodily integrity and informed consent) and equality/non-discrimination by which to assess proposed or existing laws in relation to health.

The 2015 High Court of Kenya’s judgment on the constitutionality of the so-called “Uhuru’s HIV List” is a clear example of how the application of human rights principles could enhance the validity of laws and policies adopted in relation to health. In that case, a directive was issued by the President Uhuru Kenyatta to all County Commissioners to collect data on all school-going children living with HIV/AIDS. The Court found that the directive violated children’s rights to privacy, among other things. The Court ruled that the directive and actions taken under its direction violated the Constitution, but refrained from mandating the government to adopt a certain policy or protocols that met specific criteria [44]. Moreover, as the directive had begun to be implemented, there were already children who had been harmed, and for these children the court ruled that the government must de-identify the data in such a way so that it did not link a child to their HIV status.

Such harms can be avoided when the validity of legal frameworks are considered from the outset of policymaking. For example, the development of the East African Community HIV and AIDS Prevention and Management Act of 2015 provides an illustration of a legal framework that was developed using an HRBA and which seeks to promote the rights of persons living with or affected by HIV, even overriding national laws that contain discriminatory clauses [45]. The inclusive and democratic process for developing the law, which took 7 years of extensive consultations with stakeholders, including persons living with HIV, sex workers, men who have sex with men (MSM), drug users, health care workers, parliamentarians, civil society groups and religious leaders, not only exemplifies how HRBAs promote concern for and empower marginalized communities. From the perspective of health policy and governance, the process was critical to both the law’s legitimacy as well as that of the programs that were launched within its parameters.

## Fair financing

The UN Committee on Economic, Social and Cultural Rights, which interprets the core formulation of the right to health under international law, calls for national governments to ensure that health facilities, goods, and services are, among other things, physically accessible and affordable on the basis of non-discrimination [8]. As both scholars and WHO Task Forces have pointed out, non-discrimination and equity in access to services requires equity in financing [46, 47]. According to WHO, “UHC is centrally concerned with both access to services and financial risk protection… progress toward UHC therefore requires reform of the health financing system” [47].

Indeed, it is now widely accepted that equity in access, as required under the right to health, can only be met if financing is equitable [7, 8, 20, 48, 49]. There is a governmental obligation not to treat people in accordance to their ability to pay, but in accordance to the needs that must be met to enable lives of dignity [47, 48, 50]. The UN Committee that provides interpretations the right to health under the International Covenant on Economic, Social and Cultural Rights (CESCR and ICESCR, respectively) has noted that equity demands that poorer households should not be disproportionately burdened with health expenses as compared to richer households [8]. The UN Special Rapporteur on the right to health explains, “Universal health coverage consistent with the right to health requires establishing a financing system that is equitable and pays special attention to the poor and others unable to pay for health care services, such as children and adolescents” [7].

Although all needs cannot be met in any health system, and many cannot be met immediately but only progressively in the contexts of East Africa, defining the contours of what the government is responsible for providing as an entitlement—as part of a right to health --calls for a fair process for meeting population health needs that does not rely solely on market mechanisms that would disproportionately burden and exclude the poor [47, 48, 51]. Indeed, a robustly understood HRBA requires substantial solidarity in financing, which in turn requires redistribution to level the playing field, and given their importance in the context of East Africa, effective regulation of private actors, as called for by CESCR: “States parties should … ensure that the private business sector … considers the importance of the right to health in pursuing their activities” [8].

Establishing fair financing would require tectonic shifts to the health reforms being undertaken in the region. As it stands, given the extremely low tax base common throughout the region, relying on preexisting employment schemes would undermine efforts at fair financing. In the EAC, only 36% of health care funding comes from domestic sources composed of 20% from the member states and 16% from private insurance. The remaining 63% of funding comprises 28% from out-of-pocket spending, and 35% from external sources [52].

The risk pooling pre-payment schemes that are being designed as statutory deductions for those who are registered with national health insurance schemes, heavily target those in formal employment while making little effort to address individuals employed in the informal sector [53]. Yet, in Uganda an estimated 30% of workers are in the formal sector [54]; in Kenya only an estimated 18% of people in the labor force are in the formal sector [55]; and in Rwanda an estimated 33% of employees are in the formal sector [56]. Thus, access to care for those employees—and for their children and dependents—is based not on need but on one family member’s employment status and insurance coverage, a situation which has been found to contravene constitutional and other commitments to equality by courts as well as government policy makers in other regions [50, 57].

In terms of external sources, the Global Financing Facility (GFF) has provided a $30 million grant for Reproductive, Maternal, Newborn, Child and Adolescent Health (RMNCAH) to Uganda, constituting 7% of total government health spending [58], a $40 million grant for RMNCAH to Kenya [59], and a $40 million grant for RMNCAH to Tanzania [60]. But it should be noted that targeted aid efforts do not obviate the need for more robust and equitable domestic financing. Further, in the past aid aimed at abolishing users’ fees to expand maternal-child services for indigent patients in several East African countries have produced perverse effects in practice when policies have not been aligned with programs, and where health facilities are often not well-equipped or staffed to deal with the increase in number of patients, compromising the quality of services rendered [61]. For example, in 2015, the High Court of Kenya addressed discrimination and abuses experienced by women in public maternity hospitals, and ordered compensation for two women who were unconstitutionally detained at the Pumwani Maternity Hospital for inability to pay despite the policy of free maternal care [62].

Indeed, there is a substantial risk in East Africa that UHC may devolve into coverage that does not translate into equal effective access to care in practice, unless greater attention is paid to fair financing. Adopting an HRBA provides principles to guide such a process.

## Priority setting: process and criteria matter

To advance the Global Strategy, and to achieve UHC, which is SDG Goal 3.8, countries in the region, as elsewhere, will need to make important choices. While the right to health requires states to pursue the highest attainable standard of health, it does not eliminate the need for states, rich and poor, to make choices about how to finance and what to finance within the health system. The right to health does require the process and criteria for priority setting to be equitable, non-discriminatory, participatory, accountable and transparent [8, 20].

First, states need to define which services they consider as priorities, which involves difficult trade-offs. For example, priority may be placed upon cost-effectiveness of interventions, but also may target severe diseases or disadvantaged populations, and should provide financial risk protection from the impoverishing impact of ill-health. Countries will need to establish such a list of priority services, and periodically update it every few years [47].

**Fig. 2:**
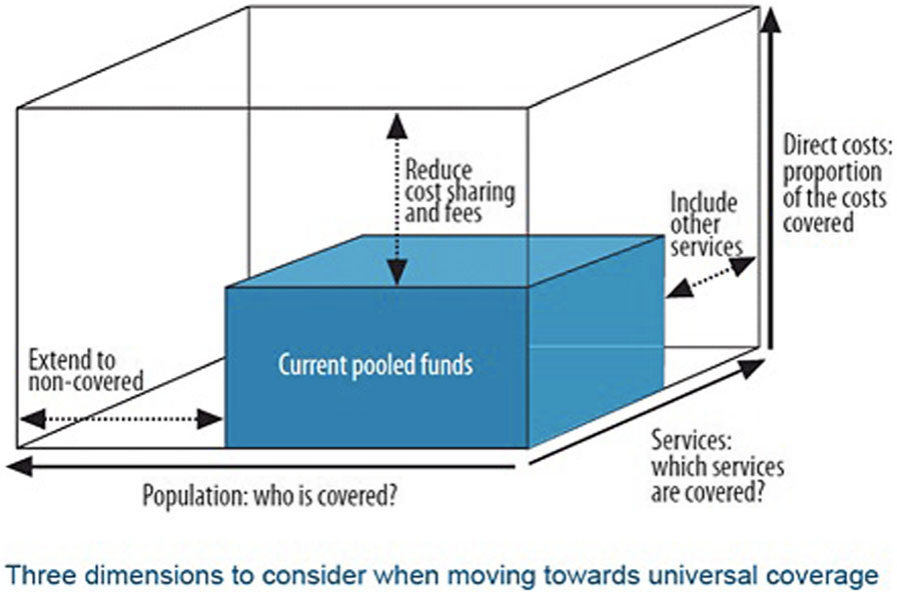
Universal coverage - three dimensions

Second, countries in the region will need to make choices regarding the implementation of priorities, as illustrated in Fig. 2 [63]. Governments have limited pooled funds for health care, so in moving towards UHC governments will have to consider 1) whether to expand coverage to those currently not covered, 2) what proportion of the costs to cover, and 3) which services to cover.

Here, governments can choose to include more priority services in the essential package, or expand coverage of existing priority services to non-covered populations, or reduce out-of-pocket payments for existing priority services. Embracing and pursuing the Global Strategy does not obviate the need for such trade-offs. For example, countries may need to choose between increasing the coverage of skilled birth attendance to all rural populations, or reducing copayments for antibiotic treatment of children with pneumonia. “The option they choose to do *first* may have far-reaching consequences for the level and distribution of health in the country, and of financial risk protection. Countries need to address these decisions on a recurrent, ongoing, basis” [64].

If health is considered a right, either under the Constitution or legislation, and the health system is understood as part of democratic governance in an HRBA, these questions are not merely technical ones, but require ensuring that citizens are active participants in the decisions that affect their health, including the definition of the contours of what their right to health includes [7]. Thus, to the extent possible, priority setting cannot be done merely on the basis of credible clinical and cost-effectiveness evidence, but must entail a legitimate democratic process. [8, 51]. As stated in the MDG Task Force on Child and Maternal Health, “Health claims—claims of entitlement to healthcare and enabling conditions—are assets of citizenship. Their effective assertion and vindication through the operation of the health system helps build a human rights culture and a stronger, more democratic society” [65].

Yet, governments in the East African region have not demonstrated openness to meaningful citizen participation in setting priorities UHC. For example, REACT, an EU funded five-year intervention that described and evaluated district-level priority-setting upon in one district each in Tanzania, Kenya and Zambia found “a high need to promote new approaches to priority-setting processes that take into account” “a broader range of relevant values, such as trust, equity, accountability and fairness” [66].

As scholars have pointed out at the global level, “sexual and reproductive rights may be systematically neglected in many essential service packages” [67]. But even those exercises intended to set priorities under the Global Strategy in the region have not been subject to robust participation. For example, “consensus-building exercises” in Uganda appear to have presented “consultations” on a predetermined package of “low-cost” interventions including voucher projects and results-based financing, which allowed the government to “complete key strategies in time” for the GFF investment deadline [68]. Similarly, Tanzania has adopted priorities without meaningful citizen participation [69]. Yet, such decisions as placing priority upon the “development of a systematic scale-up plan for long-acting and permanent family planning services to ensure marginalized groups are reached” affect people’s most intimate decisions about their life plans and reproductive lives, and therefore their dignity. In an HRBA, such priority-setting would require inclusion of the voices of people affected, including marginalized and vulnerable populations, such as people living with HIV, and persons with disabilities.

Admittedly, incorporating processes that enable people to meaningfully participate in some of the ranking and criteria for priorities (not choosing individual services) is time-consuming, expensive and extremely challenging, given information asymmetries and diverse interests in the health sector. Indeed, “governments by discussion” are never easy, as philosophers from the Greeks to John Stuart Mill realized [70, 71]. Yet, these processes are both necessary from a principled approach to human rights, and are more likely to result in decisions that are more socially legitimate. The WHO has reported that participation of women in the design, implementation, management and evaluation of community health systems is associated with “improved health and health-related outcomes,” including significant improvements in mortality rates for children and positive outcomes for contraceptive use and service uptake [72].

Small-scale examples from the region show the transformative potential of meaningful participation in HRBAs. For example, in 2015, the Commission for the Implementation of the Constitution in Kenya conducted a participatory research and implementation project that entailed multi-stakeholder workshops, in-depth surveys with users, and capacity-strengthening with providers over months. It resulted in revised service charters in the three counties, greater capacity of providers as well as awareness of patients’ rights, and a blueprint for further steps based on the granular realities of each specific context [73].

## Oversight mechanisms: from monitoring and review to meaningful accountability

Accountability is perhaps the sine qua non of an HRBA [13, 74], and what sets it apart from other approaches to global health focused on equity. Human rights identifies health system users as active claims-holders and governments and other actors as duty-bearers. Monitoring efforts to assess effectiveness, with reliable and, to the extent possible, disaggregated data (to reveal disparities, and potential discrimination) is essential to enabling accountability. Yet monitoring data alone is insufficient; oversight mechanisms are needed to regulate power imbalances that plague the sector.

**Fig. 3:**
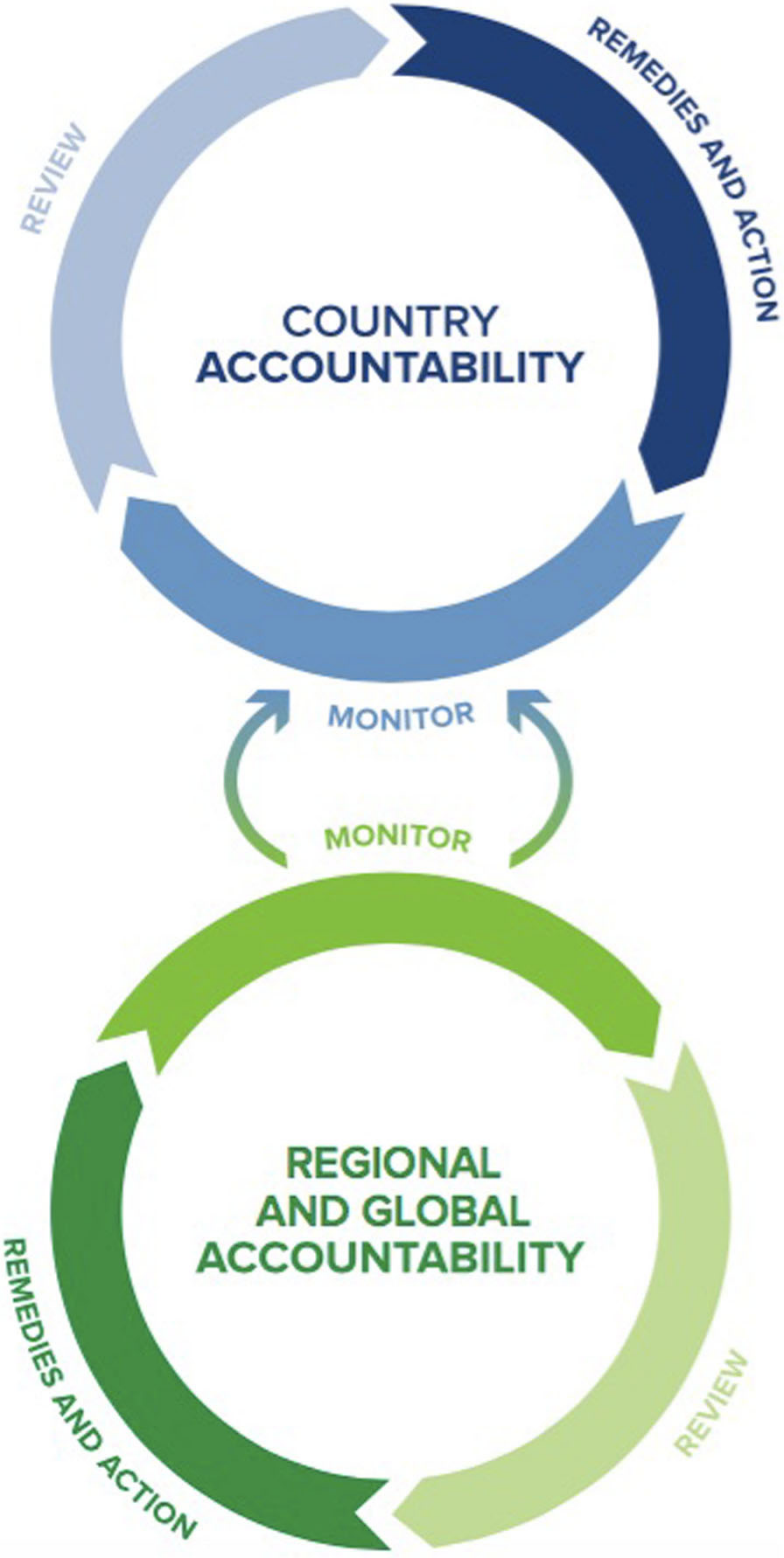
IAP accountability framework

The accountability framework adopted by the UN Secretary General’s Independent Accountability Panel for the Global Strategy illustrates how monitoring, review, action, and remedies all form part of an HRBA to accountability, as shown in Fig. 3 [14]. Drawing from authoritative interpretations of international law [8, 9, 75], the monitor, review, action and remedy framework operates at both the national level, but also at the regional or international level [14].

In addition to monitoring data, the IAP framework calls for a process of continuous review at both the local and global levels, by actors ranging from independent agencies to civil society. It also calls for oversight institutions to act in response to the review mechanisms by enforcing sanctions and providing incentives dependent upon compliance. Finally, it calls for remedies in the form of political action, social engagement, and judicial enforcement of health-related rights [76]. These actors, including parliamentary bodies, independent judiciaries and national human rights institutions with appropriate mandates and capacity, all can and should play key roles in ensuring that the health system is enshrining the normative commitments to equal dignity set out in international human rights law and national constitutions—in both law and practice [76].

In particular, independent judiciaries can play important roles in ensuring the values reflected in health systems are consistent with democratic commitments, and that health systems are understood as subject to the rule of law [20, 77]. Courts in the region are indeed already playing this role to some extent, which can re-orient norms and policies. For example, as mentioned above, a 2015 High Court of Kenya’s judgment ordered an end to discrimination and abuses experienced by women in public maternity hospitals, and compensation for two women who were unconstitutionally detained at the Pumwani Maternity Hospital for inability to pay [62]. In a 2016 case in Uganda, concerning the failure of the Mulago National Referral Hospital to protect mothers from having their babies stolen, the High Court of Uganda ordered access to civil society to monitor implementation of orders and make reports to the Court [78]. In a 2011 case in the Constitutional Court of Uganda, the court found that access to obstetric care services and human resources for maternal health constitute a part of the right to life guaranteed in Article 22 of Uganda’s Constitution [79].

Such remedies should not be confused with addressing malpractice; they are institutional remedies that are essential to not merely sanction violations but to prevent future abuses, ensure fidelity to constitutional and international norms, and subject health policies and programs to independent scrutiny of reasonableness. Requiring governments to publicly justify policies, and whether they are implemented appropriately, is as essential in health systems as in any other area of democracy [80].

## Conclusion

There is increasing rhetoric about the importance of human rights to achieving the Global Strategy and the SDGs relating to health at global levels. Governments in East Africa are also acknowledging the link between health and rights [81]. Yet there continues to be a gap between this rhetoric and the reality of what applying human rights to efforts to achieve UHC would entail, and there is a real risk that the increased rhetoric of human rights replaces meaningful rights-based action.

We have argued here that rights are fundamentally about the regulation of power, and to understand health as a right is to understand that patterns of health and ill-health require democratic controls over power as they do in other arenas. Within the scope of this article it has not been possible to consider every aspect of HRBAs but specifically, we have argued that legal and policy frameworks play a critical role in establishing parameters for people to exercise their health-related rights and should be incorporated into multi-sectoral planning and situational analyses. Second, if health is a right, fair financing needs to divorce meeting essential health needs necessary for people’s dignity from ability to pay, which will require substantial reorientation of financing plans for the region. Third, meaningful participation in an HRBA requires stakeholders to be involved in voicing their needs when priorities are set, and the contours of essential packages and health reforms are undertaken. Finally, oversight mechanisms should include not only monitoring and evaluation within the health sector, but also meaningful remedies that call for greater public justification and accountability with respect to fundamental normative commitments as well as alignment of programmatic practice with policies and laws.

The EAC’s “Open Health Initiative”—aimed at harmonizing the health systems of member countries to promote UHC—provides an ideal opportunity for policy makers to create broader dialogues about the role of health systems in fostering better democratic governance [82, 83]. Policy makers at the EAC and national level, together with development partners, can enhance the equity, transparency and accountability of ongoing health reforms, as well as their democratic legitimacy, by taking human rights principles seriously. But ultimately conquering health rights and constructing inclusive, fair health systems will require ongoing struggle and vigilance by civil society, just as it does elsewhere.

## Abbreviations

CESCR: Committee on Economic, Social and Cultural Rights
EAC: East African Community
GFF: Global Financing Facility
HRBA: Human rights-based approach
IAP: International Accountability Panel
ICESCR: International Covenant on Economic, Social and Cultural Rights
MDG: Millennium Development Goal
MMR: Maternal mortality ratio
MSM: Men who have sex with men
PEPFAR: President’s Emergency Plan for AIDS Relief
RMNCAH: Reproductive, Maternal, Newborn, Child and Adolescent Health
SBA: Skilled birth attendance
SDG: Sustainable Development Goal
UHC: Universal Health Coverage
WHO: World Health Organization

## Footnotes

1 East Africa is a geographically defined region comprised of up to 20 states located on the East coast of Africa, including at minimum the East African Community states.

2 The East African Community is a regional intergovernmental organization comprised of six member states: Tanzania, Uganda, Burundi, Kenya, Rwanda and South Sudan.

3 All opinions expressed in this article are personal and do not reflect official positions of the IAP.
